# Cooking with Wood May Fuel Low Birth Weight: Kitchen Smoke Puts Babies at Risk

**DOI:** 10.1289/ehp.116-a173a

**Published:** 2008-04

**Authors:** Cynthia Washam

The etiology of low birth weight (LBW; defined as weighing less than 2.5 kg at birth) is complex, with demographic, nutritional, reproductive, and socioeconomic factors each potentially playing a role. Inhaled tobacco smoke is the leading cause of LBW in industrialized countries, and inhaled smoke from the world’s most widely used cooking fuel, wood, can impair fetal growth much the same way. A team of researchers therefore launched a population-based study to examine the risk of LBW specifically in relation to use of wood fuel during pregnancy **[*EHP* 116:543–549; Siddiqui et al.]**. They found that maternal exposure to pollutants from wood smoke increases the risk of LBW, which is linked with myriad health problems including nutritional deficiencies, impaired psychomotor development, and chronic disease.

Tobacco smoke and wood smoke work in two ways to thwart fetal development. One occurs when carbon monoxide combines with hemoglobin to cross the placenta. This causes hypoxia, or a decreased oxygen supply to tissue, which limits the ability of the placenta to transfer nutrients to the fetus. The other occurs when inhaled particulate matter from smoke impairs fetal growth by damaging cells through oxidative stress.

The team of U.S.- and Pakistan-based researchers studied births in the latter country, where more than half the population cooks with wood and the 19% LBW rate is among the world’s highest. The researchers studied 634 women who gave birth from 2000 through 2002 in the poor, semirural community of Rehri Goth. Interviewers collected data on the mothers’ cooking habits and family demographics. The researchers also obtained pregnancy and delivery data from the mothers’ and infants’ medical records.

Women who used wood fuel during pregnancy had a significantly higher risk of delivering LBW babies than those who cooked with natural gas—23% versus 15%. More time spent cooking was linked with increased LBW risk in wood users but not natural gas users. Wood users were poorer than users of natural gas; more of them lived in crowded, run-down houses; and they were more likely to be illiterate. Wood users also tended to weigh less than natural gas users.

Although such socioeconomic factors may play a greater role in birth outcomes, cooking fuel is one factor that is relatively amenable to change. The authors now propose studies of the health impact of smoke-free stoves. The World Health Organization has predicted that if all Pakistani households cooking with wood converted to cleaner fuels, the incidence of LBW would fall from the current rate of 19% to just below the 15% target rate set by the organization.

## Figures and Tables

**Figure:**
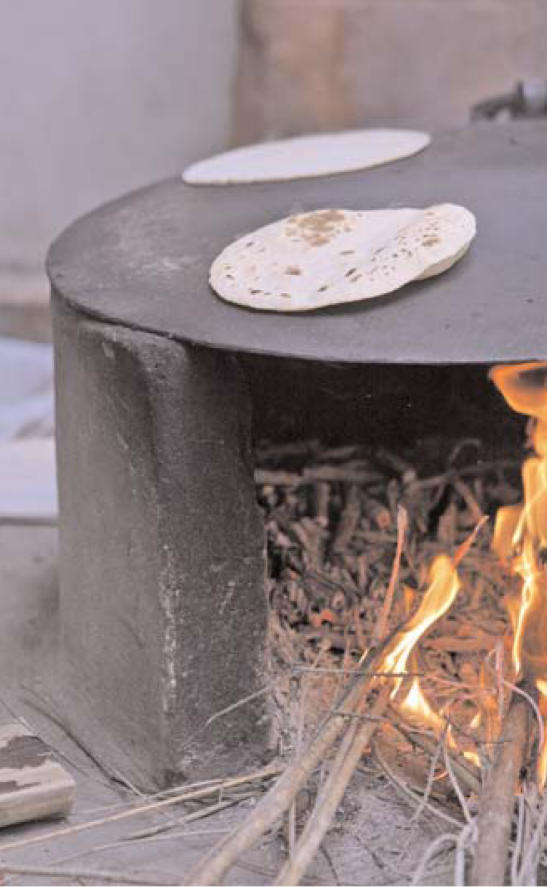
Wood is the cooking fuel of choice for 53% of Pakistani households

